# The Effect of Isovolemic Hemodilution with Oxycyte®, a Perfluorocarbon Emulsion, on Cerebral Blood Flow in Rats

**DOI:** 10.1371/journal.pone.0002010

**Published:** 2008-04-23

**Authors:** Zhong-jin Yang, Chrystal D. Price, Gerardo Bosco, Micheal Tucci, Nagwa S. El-Badri, Devanand Mangar, Enrico M. Camporesi

**Affiliations:** 1 Department of Anesthesiology, SUNY Upstate Medical University, Syracuse, New York, United States of America; 2 Department of Anesthesiology, University of South Florida, Tampa, Florida; 3 Department of Physiology, University of Chieti, Chieti, Italy; Women's and Children's Hospital, Australia

## Abstract

**Background:**

Cerebral blood flow (CBF) is auto-regulated to meet the brain's metabolic requirements. Oxycyte® is a perfluorocarbon emulsion that acts as a highly effective oxygen carrier compared to blood. The aim of this study is to determine the effects of Oxycyte® on regional CBF (rCBF), by evaluating the effects of stepwise isovolemic hemodilution with Oxycyte® on CBF.

**Methodology:**

Male rats were intubated and ventilated with 100% O_2_ under isoflurane anesthesia. The regional (striatum) CBF (rCBF) was measured with a laser doppler flowmeter (LDF). Stepwise isovolemic hemodilution was performed by withdrawing 4ml of blood and substituting the same volume of 5% albumin or 2 ml Oxycyte® plus 2 ml albumin at 20-minute intervals until the hematocrit (Hct) values reached 5%.

**Principal Findings:**

In the albumin-treated group, rCBF progressively increased to approximately twice its baseline level (208±30%) when Hct levels were less than 10%. In the Oxycyte®-treated group on the other hand, rCBF increased by significantly smaller increments, and this group's mean rCBF was only slightly higher than baseline (118±18%) when Hct levels were less than 10%. Similarly, in the albumin-treated group, rCBF started to increase when hemodilution with albumin caused the CaO_2_ to decrease below 17.5 ml/dl. Thereafter, the increase in rCBF was accompanied by a nearly proportional decrease in the CaO_2_ level. In the Oxycyte®-treated group, the increase in rCBF was significantly smaller than in the albumin-treated group when the CaO_2_ level dropped below 10 ml/dl (142±20% vs. 186±26%), and rCBF returned to almost baseline levels (106±15) when the CaO_2_ level was below 7 ml/dl.

**Conclusions/Significance:**

Hemodilution with Oxycyte® was accompanied with higher CaO_2_ and PO_2_ than control group treated with albumin alone. This effect may be partially responsible for maintaining relatively constant CBF and not allowing the elevated blood flow that was observed with albumin.

## Introduction

Perfluorocarbon solutions (PFCs) are biochemically inert organic compounds with a high affinity for oxygen and CO_2_ that can improve tissue oxygenation when used with high inspiratory concentrations of oxygen [1]. PFCs have long been proposed as potential blood substitutes to reduce the need for allogeneic blood transfusions in a variety of clinical situations. Although PFCs were introduced in the 1970s, their applications and safety profiles have yet to be defined [1], [2].

The impact of PFCs on cerebral function is still under investigation, including its effects on cerebral blood flow (CBF). It is well known that hemodilution, characterized by decreased hematocrit (Hct), lowered circulating oxygen content (CaO_2_), and decreased blood viscosity can increase CBF [3]–[7]. Results of animal studies suggest that PFC administration decreases cerebrovascular resistance and blood viscosity, thereby increasing CBF [8], [9]. In most cases, increased CBF reflects increased metabolic demand or decreased arterial oxygen content (CaO2), and may indicate a need to increase cerebral oxygen delivery.

Because of the tiny particle size, PFC emulsions readily perfuse in the microcirculation, resulting in augmented oxygen delivery to the tissues more effectively than oxygen-carrying blood cells in the large vessels [9]. Because of this simple physical transport system, PFC emulsions load and unload oxygen twice as fast as does hemoglobin [10]. Although the exact capacity of oxygen delivery by PFC compared to Hb is not known, several reports indicate that oxygen delivery potential of a 2.7 g/kg dose of PFC is approximately equivalent to 4 g/dL [Hb] [11], [12]. Although the clinical importance of PFC-induced increases in CBF is unclear, animal studies suggest that PFCs may be neuroprotective in patients with traumatic and ischemic brain injury [13]–[16].

Oxycyte® is a perfluorocarbon emulsion that has the ability to dissolve large amounts of oxygen, and has been shown to be a safe blood alternative in Phase I clinical trials. Oxycyte® particles can be efficiently carried to tissues perfused at critically low flow rates, while eliciting no vasoconstriction or associated pharmacological disadvantage that prevents tissues from utilizing this oxygen. In a model of permanent middle cerebral artery occlusion in rats Oxycyte® has been shown to decrease ischemic brain damage [17]. A recent study showed that early administration of Oxycyte® immediately after the onset of vascular occlusion exerted a neuroprotective effect in these rats [18].

Several studies showed that PFC emulsions have higher oxygen carrying capacity compared to Hb, and are able to deliver twice the oxygen to the tissues when combined with hyperoxic ventilation [12], [9]–[11]. Furthermore, Adminsitration of PFC emulsions has been well tolerated by infused animals and has no negative effects on microvascular perfusion [12], [19]. To determine whether oxygen utilization by cerebral tissue is modified when Oxycyte® is used for hemodilution, the present study was performed to evaluate the effects of stepwise isovolemic hemodilution with Oxycyte® on regional CBF (rCBF) in experimental rats.

## Methods

### Experimental Animals

The proposed study was approved by the Institutional Committee for the Humane Use of Animals at Upstate Medical University, Syracuse, New York 13210, in accordance with the guidelines established by the National Institutes of Health. The work was performed in the Department of Anesthesiology Basic Science Laboratory at SUNY Upstate Medical University. Pathogen-free male Sprague-Dawley rats weighing between 350 to 400 g (Taconic Farm, Germantown, NY) were used. Rats were kept in holding wire mesh cages for a week to acclimate them to the study and environmental conditions. Standard rat chow (Diet #5008, Ralston Purina, St. Louis, MO) and tap water were provided *ad libitum*.

### Surgical Procedures

All rats were anesthetized by intramuscular injection of a rodent anesthetic mixture containing ketamine and xylazine (100:10 mg/ml) at a dose of 1.0 ml/ kg. The neck, head and both groin regions were shaved and the skin was prepared with 10% solution of Povidone-Iodine (Betadine, Purdue Products L.P, Stamford, CT). Body temperature was monitored with a rectal probe (Mon-a-therm 6500, Mallinckrodt Medical, St. Louis, MO) and maintained at approximately 37.0±0.5°C with a heating pad and light.

Tracheostomy was performed and the rats were mechanically ventilated (Harvard Apparatus, South Natick, MA) with 100% oxygen at a frequency of 40 to 60/min and a tidal volume of 3.0 ml. End tidal CO_2_ pressure was kept close to 40 mmHg by varying ventilation settings. Anesthesia was maintained with isoflurane (Minrad inc., Bethlehem, PA) at an inspiratory concentration of 1.2%. Both femoral arteries and veins were cannulated with polyethylene tubing (ID 0.58mm, Becton-Dickinson Co., Parsippany, NJ). Under anesthesia, the rats' hair was shaved on the ventral neck from the right groin to the mid-thigh, and the area swabbed with iodine solution. A 1.5 cm incision was made in the midline of the inner surface of the leg to expose the femoral artery and vein. The right femoral artery and vein were carefully separated with a pair of hemostats and a 5 cm silk tie was tied loosely around the vessels. A small incision was made and the catheter was inserted and secured with silk tie. The skin was then closed. The left femoral artery and vein were cannulated in the same manner. The right artery catheter was used to monitor blood pressure, and the left artery catheter to collect samples. The left venous catheter was used to infuse phenylephrine and the right venous catheter was used to replace collected blood volume according to the experimental protocol.

### Regional Cerebral Blood Flow Measurement

After skin closure, the rat was placed prone on a stereotactic frame (David Kopf Instruments, Tujunga, CA). A midline skin incision over the skull was performed, and a small burr hole was drilled. A 26 G, single-fibre laser-Doppler flow (LDF) probe (DP4, Moor Instruments Inc, Wilmington, DE), connected to a monitor (Moorlab server, Moor Instruments Inc., Wilmington, DE) was inserted into the right striatum. The stereotactic coordinates from the bregma were: medial lateral, 4 mm from the middle line and dorsal–ventral, 4 mm ventral from the surface of the dura [20]. The LDF device was calibrated with a standard calibration solution provided by the manufacturer. The probe was allowed to stabilize for 20 minutes before a baseline signal was obtained, which represented baseline (ie, 100%) rCBF. Changes in rCBF were expressed as the percentage change from baseline.

### Stepwise Isovolemic Hemodilution

All rats received 0.15 ml intravenous heparin (500 IU ml^−1^) to prevent blood from coagulating in the catheters. The baseline cmean arterial pressure (MAP), arterial blood gases (ABG), Hct and rCBF were obtained. The rats were randomized to Oxycyte®-treated (n = 7) and albumin-treated (n = 7) groups. Isovolemic hemodilution was performed by removing 4 ml of blood at a rate of 0.5–1 ml/min and then substituting the same amount of 5% albumin solution (Baxter Healthcare Co., Glendale, CA) in albumin-treated control rats or 2 ml Oxycyte® (Synthetic Blood International Inc, Costa Mesa, CA) plus 2 ml 5% albumin in Oxycyte®-treated rats. Oxycyte® consists of a perfluoronated C-10, cyclohexane compound [60% (wt/vol)] emulsion, with egg yolk lecithin as the emulsifier. The nominal diameter of emulsion particles was 0.2 µm. The oxygen dissolving capacity of Oxycyte® is (43) vol% (43 ml O_2_/100 ml pure PFC) at 37°C and 760 mmHgO_2_.

After the first hemodilution step, Hct was determined again, and ABG was analyzed. The next steps of hemodilution were calculated to serially approach target Hct of 25%, 20%, 15% and 12%. Hemodilution was continued stepwise at 20-minute intervals. In the Oxycyte®-treated group, the target concentration of Oxycyte® was between 7% and 10%. MAP was maintained above 60 mmHg by continuous infusion of phenylephrine (Gensia Sicor Inc. Irvine, CA) up to15 mcg/h. The experiment was completed when Hct values reached 5%. The oxygen content (CaO_2_) was calculated with the following equation:
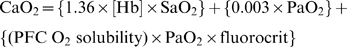



Hb: hemoglobin; CaO_2_ : arterial oxygen content; SaO_2_ : arterial saturation of Hb with oxygen (%); PaO_2_: arterial oxygen partial pressure; fluocrit: arterial Oxycyte concentration.

Hb concentration was calculated from the Hct as follows: Hb (g/dl) = Hct×0.34 (Mean cell Hb concentrations were assumed to be comparable in all rats). After hemodilution, supplemental phenylephrine infusion was required in order to maintain blood pressure within 10% of control especially after 2–3 hemodilution steps.

### Statistical analysis

All data were presented as Mean±SD. Changes within each group were analyzed using the analysis of variance (ANOVA) for repeated measures. Differences between the groups at any time point were tested using two way ANOVA followed by the Bonferroni test as a post–hoc test. P<0.05 is considered as a statistically significant.

## Results

### Oxycyte® hemodilution and circulating oxygen content as determined by hemodynamic parameter

The rat groups treated with Oxycyte® showed no significant difference in MAP, PaCO_2_, or pH when compared to rat group treated with albumin alone at the end of the experiment (Hct less than 10%, Table 1). However, the PaO_2_ was significantly higher in rats hemodiluted with Oxycyte® than in rats hemodiluted with albumin. Interestingly, a higher infusion rate of phenylephrine was required to maintain MAP in the albumin group, but within a reasonably low infusion rate. Furthermore, no significant change in body temperature was observed between the two groups even after a prolonged experiment over a duration of 5 hours (data not shown).

**Table 1:** Hemodynamics and blood gas data at the point of maximum hemodilution (n = 7 in each group)

	Oxycyte®	Albumin	p
PaCO_2_ (mmHg)	37.19±0.50	38.01±0.74	0.351
PaO_2_ (mmHg)	502.1±10.46	419.93±15.92	<0.001
pH	7.369±0.006	7.376±0.006	0.418
MAP (mmHg)	74.32±0.87	73.55±0.89	0.538
Phenylephrine (mcg/h)	1.98±0.20	3.06±0.34	0.002
weight (g)	349± 13	348±11	0.928

Data as mean±SD (standard deviation of the mean)

### Oxycyte® and calculated arterial oxygen content

**Figure 1:**
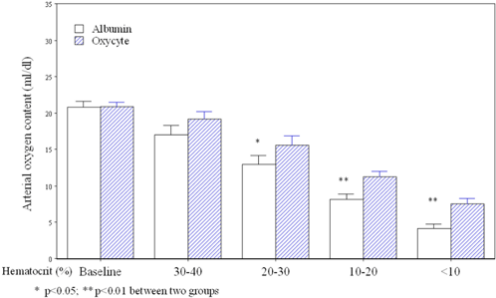
The changes of calculated arterial oxygen content during acute isovolemic hemodilution with Oxycyte® or albumin. Hemodilution causes a progressive decrease in Hct level. The arterial oxygen content decreases in an inverse proportion to the levels of Hct during hemodilution with either albumin or Oxycyte®. However, the arterial oxygen content is maintained at a significantly higher level during hemodilution with the Oxycyte® group than in hemodilution with the albumin group. Open bar: acute isovolemic hemodilution with albumin; Shaded bar: acute isovolemic hemodilution with Oxycyte®.

As shown in Figure 1, CaO_2_ progressively decreased, as did Hct values, during stepwise isovolemic hemodilution in both albumin and Oxycyte® treated groups. Nevertheless, hemodilution with Oxycyte® produced significantly higher CaO_2_ levels than did hemodilution with albumin; for the Oxycyte® and albumin groups (respectively): mean CaO_2_ levels were 15.6±0.5vs. 12.9±0.5 (p<0.05) at Hct level of 20–30%, 11.2±0.3 vs. 8.1±0.3 (p<0.05) at Hct level of 10–20% and 7.5±0.3 vs. 4.5±0.2 (p<0.01) at Hct level of less that 10%.

### Hemodilution and regional cerebral blood flow

**Figure 2:**
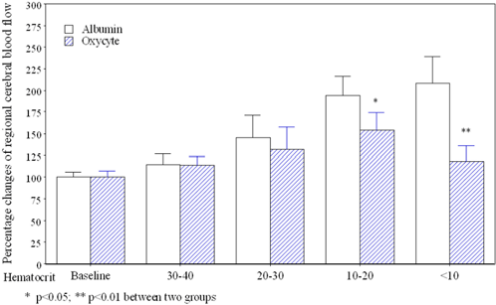
The changes of regional cerebral blood flow during acute isovolemic hemodilution with Oxycyte® or albumin. Hemodilution causes a progressive decrease in Hct level. The regional cerebral blood flow increases in an inverse proportion to the levels of Hct and doubles from baseline when Hct level is lower than 10% during hemodilution with albumin. During hemodilution with Oxycyte®, the regional cerebral blood flow increases to 150% of baseline when the Hct is between 10–20% and then decreases to about 120% of baseline when the Hct decreased to below 10%. Open bar: acute isovolemic hemodilution with albumin; Shaded bar: acute isovolemic hemodilution with Oxycyte®.

**Figure 3:**
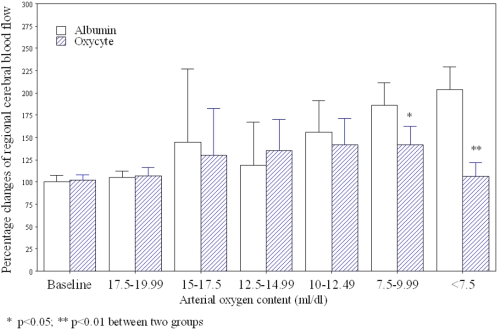
The changes of regional cerebral blood flow during acute isovolemic hemodilution with Oxycyte® or albumin. Hemodilution causes a progressive decrease in arterial oxygen content level. The regional cerebral blood flow continues to increase in an inverse proportion to the levels of arterial oxygen content during hemodilution with albumin. During hemodilution with Oxycyte®, the regional cerebral blood flow increases significantly less when arterial oxygen content decreased to below 10 dl/ml. Open bar: acute isovolemic hemodilution with albumin; Shaded bar: acute isovolemic hemodilution with Oxycyte®.

Hemodilution caused by both Oxycyte® and albumin significantly affected the relationship between rCBF and both Hct (Figure 2) and CaO_2_ (Figure 3). In the albumin-treated group, rCBF progressively increased to approximately twice its baseline level when Hct levels were less than 10%. In the Oxycyte®-treated group on the other hand, rCBF increased by significantly smaller increments, and this group's mean rCBF was only slightly higher than baseline when Hct levels decreased to less than 10%. Similarly, in the albumin-treated group, rCBF started to increase when hemodilution with albumin caused the CaO_2_ to decrease below 17.5 ml/dl. Thereafter, the increase in rCBF was accompanied by a nearly proportional decrease in the CaO2 level. In the Oxycyte-treated group, the increase in rCBF was significantly smaller than in the albumin-treated group when the CaO2 level dropped below 10 ml/dl, and rCBF returned to almost baseline when the CaO2 level was below 7 ml/dl.

## Discussion

In this study we show that acute isovolemic hemodilution with Oxycyte® maintains significantly higher circulating oxygen content than does isovolemic dilution with albumin alone. Furthermore, administration of both Oxycyte® and albumin increased rCBF after acute isovolemic hemodilution, albeit in different patterns. With albumin, rCBF increased progressively in inverse proportion to the decreased Hct and CaO_2_. Under the most extreme isovolemic hemodilution conditions, rCBF was approximately 170% higher in the albumin-treated group than in the Oxycyte®-treated group when the two groups were compared at the same concentrations of Hct and circulating oxygen content (i.e., Hct <10% and CaO_2_ <7.5 ml/dl), indicating that Oxycyte® has maintained baseline CBF. Table 1. shows that pH and PCO_2_ were kept constant in both groups.

Laser Doppler flowmeter (LDF) was used in the present study to show the increase in rCBF after acute hemodilution with Oxycyte® or albumin. As Tonnesen et al [21] have pointed out, LDF is a valuable tool for continuously measuring relative changes in rCBF; for example, under conditions of hypovolemic hemodilution and hemorrhagic shock caused by controlled hemorrhage, the LDF method may yield significantly higher relative rCBF values than the ^133^xenon injection technique. The advantage of using LDF in cerebral perfusion studies is that this technique can measure rCBF *in vivo*, continuously, for up to several hours. Harada et al have reported the reproducibility of the LDF monitoring in minimizing the risk and reducing intraanimal variability and unexpected death for rat MCAO model [22].

The increase in CBF caused by hemodilution with PFC emulsion has long been recognized; the mechanisms by which PFC emulsions increase CBF are probably multifactorial. Lower blood viscosity resulting from isovolemic hemodilution, the small particle size of the PFC emulsion and decreased circulating oxygen content are all contributing factors. Hiraga et al [23] used [14C] iodoantipyrine and quantitative autoradiographic techniques to measure local CBF in rats breathing 100% oxygen after blood-PFC exchange. The CBF increased approximately two-fold after complete blood-PFC exchange and 1.5-fold after partial exchange. The authors attributed the increase in CBF to an autoregulatory response of the central nervous system vasculature to maintain an adequate supply of oxygen to the central the nervous system tissue. Lee et al [24], [25] used a radiolabeled-microsphere technique to evaluate cerebrocirculatory responses to total perfluorocarbon (FC-43) for-blood replacement (Hct less than 1%) in anesthetized, ventilated rats breathing 100% O_2_. Exchange transfusion with FC-43 doubled the total and regional CBF, causing preferential flow increases to the cortex and cerebellum. Estimated cerebrovascular resistance fell to 50% of the pre-exchange value. Both hemodilution and hypoxia experiments produced CBF responses similar to those obtained in FC-43 animals. Their results suggested that decreased oxygen content and, perhaps, lower viscosity of the circulating fluorocarbon were responsible for increases in CBF required to maintain sufficient delivery of oxygen to the brain.

In the present study, partial Oxycyte®-for-blood exchange increased rCBF approximately 150%. However, rCBF did not further increase in response to decreased Hct and circulating oxygen content, but instead returned toward almost the baseline level, while Hct decreased to less than 10% and CaO_2_ decreased to less than 7.5 ml/dl. Increased rCBF in response to decreased circulating oxygen content is a normal physiological response of the brain. The reason for different of rCBF changes under extreme conditions between our study and others [23]–[25] is unclear; however, the different cerebral regions of measured blood flow (striatum in our study) and the different structures of PFC emulsions (Oxycyte® in our study) may be partially responsible for this discrepancy. The impact of perfluorocarbon emulsion on neural cells' structure and function warrants further investigations. The present study's findings suggest that when Oxycyte® is used for hemodilution, care should be taken to keep circulating oxygen content from becoming too low (<7.5 ml/dl) to maintain adequate oxygen tissue perfusion.

Under normal physiological conditions, increased CBF is associated with improved cerebral oxygenation. Findings from animal studies have suggested that the increased CBF and improved oxygenation may be beneficial for patients with ischemic and traumatic brain injury [13]–[16].

In the present study, the rats were anesthetized, and their physiological parameters were kept within normal ranges (e.g., normal range of PaCO_2_/pH). The increased rCBF after isovolemic hemodilution could be attributed to a cerebral autoregulatory response that services to maintain a sufficient supply of oxygen and normal cerebral functions. The reason for the smaller increase in rCBF after hemodilution with Oxycyte® than with albumin, even when the circulating oxygen content was the same, is unknown. Although calculated circulating oxygen content is the same, the oxygen availability of red blood cells (RBCs) and PFC emulsions may be different. The average particle diameter of PFC emulsions is about 0.2 micron, compared to 5–7 microns for RBCs. An emulsion of PFC, therefore, can further enhance tissue oxygenation by filling the plasma gaps between RBCs and blood vessel walls and by perfusing constricted microcapillaries too small for RBCs to pass, thereby improving local tissue oxygenation [26]. The smaller increase in rCBF after hemodilution with Oxycyte® may therefore account for the greater amount of available circulating oxygen in the Oxycyte® recipients.

As a recent study examined the rheologic effects of PFC emulsion in the presence of clinically used volume expanders. The authors observed that hemodilution with a new perfluorocarbon emulsion significantly increased plasma and whole-blood viscosity. Viscosity values were maintained at physiologic range when human albumin-PFC mixture was used. Red blood cells deformability was also observed unchanged [27]. It has also observed that hemodynamic stability was better maintained when colloid solutions, including albumin were used in hemodilution [19]. In the present study, albumin was added to Oxycyte to minimize possible increased blood viscosity caused by Oxycyte.

PFCs have been designed to substitute for blood transfusion used in lower hematocrit status (hemodilution, hemorrhage), therefore so far there is no study to look into the effect of PFCs on CBF under normal hematocrit status. In our study, stepwise hemodilution was performed, there was no significant effect of Oxycyte on rCBF when Hct was only minimally (Hct 30–40) reduced, suggesting Oxycyte per se (in small doses) may have no significant effect on CBF when given to normal Hct status. Few studies in which second generation PFCs were added to the pump prime have demonstrated improved tissue oxygenation and increased cerebral blood flow [28]–[31].

One of the limitations in the present study was that moderate phenylephrine infusion was necessary to maintain the hemodynamic stability during acute hemodilution. Phenylephrine, a pure alpha-adrenergic agonist, produces both venous and arterial constriction. Excessive vasoconstriction and increased systemic vascular resistance may decrease cardiac output, but the direct or indirect effects of phenylephrine infusion on rCBF are unknown. In the present study, rCBF was measured in the striatum only; therefore, our data do not reflect blood flow changes that might have occurred in other regions of the brain.

We therefore conclude that although Oxycyte® and albumin administration both were accompanied by an increase of rCBF following acute isovolemic hemodilution, each does so with different variability. The different patterns of increased rCBF could be due to the ability of Oxycyte®, as a true oxygen therapeutic, to maintain higher levels of circulating oxygen content under similar physiological conditions.
